# What is a Hotspot Anyway?

**DOI:** 10.4269/ajtmh.16-0427

**Published:** 2017-06-07

**Authors:** Justin Lessler, Andrew S. Azman, Heather S. McKay, Sean M. Moore

**Affiliations:** 1 Department of Epidemiology, Johns Hopkins University, Baltimore, Maryland; 2 Medecins Sans Frontieres, Geneva, Switzerland

## Abstract

The importance of spatial clusters, or “hotspots,” in infectious disease epidemiology has been increasingly recognized, and targeting hotspots is often seen as an important component of disease-control strategies. However, the precise meaning of “hotspot” varies widely in current research and policy documents. Hotspots have been variously described as areas of elevated incidence or prevalence, higher transmission efficiency or risk, or higher probability of disease emergence. This ambiguity has led to confusion and may result in mistaken inferences regarding the best way to target interventions. We surveyed the literature on epidemiologic hotspots, examining the multitude of ways in which the term is used; and highlight the difference in the geographic scale of hotspots and the properties they are supposed to have. In response to the diversity in the term's usage, we advocate the use of more precise terms, such as “burden hotspot,” “transmission hotspot,” and “emergence hotspot,” as well as explicit specification of the spatiotemporal scale of interest. Increased precision in terminology is needed to ensure clear and effective policies for disease control.

Over the past decade there has been increasing interest in geographic disease “hotspots,” particularly for infectious diseases. This includes calls to target hotspots in public health practice.^1^–^3^ For example, at a 2013 meeting of the Reference Group on Estimates, Modeling, and Projections, the Joint United Nations Program on human immunodeficiency virus (HIV)/acquired immune deficiency syndrome (AIDS) focused on the urgent need to prioritize interventions that target geographic areas of concentrated HIV epidemics, recommending the use of novel geospatial “hotspot” analyses to inform resource allocation and programmatic goals.^4^ The third phase of the U.S. President's Emergency Plan for AIDS Relief also calls for data-driven geographic targeting of interventions, though the term “hotspot” is not used.^5^ Likewise, a November 2015 Consultative Meeting on Strategic Approaches for Cholera Preparedness and Response in the World Health Organization-Eastern Mediterranean Region recognized the mapping of cholera “hotspots” as an integral component of a regional framework for cholera control. Similar recommendations to focus on hotspots has been part of strategic plans for Ebola,^6^ malaria,^7^ and emerging pandemic threats.^8^

Despite its increasing use in policy documents, the term “hotspot” is vague and rarely defined precisely. This ambiguity can generate confusion. For instance, at the December 2014 Cholera Round Table in Kinshasa the focus was on targeting cholera hotspots; but attendees expressed considerable confusion about precisely what a hotspot was and how it differed from previous concepts like cholera “sanctuaries.” Such confusion can have significant repercussions, potentially leading to misinterpretation of policy recommendations or misallocation of resources in countries that can ill afford to do so. Hence, clarity and precision are needed in the use of this evocative term.

This article aims to provide a perspective on the use of the term “hotspot” in the public health literature, highlighting subtle differences in usage that may cause confusion in scientific and policy discussions. The origins and broader uses of the term provide some context for understanding the different ways “hotspot” is used in infectious disease epidemiology and public health. In the medical literature, the term hotspot was first used to describe a region of inflamed or irritated skin.^9^ This usage as a term of increased intensity continues in radiology,^10^,^11^ and is analogous to references to hotspots as areas of increased risk or incidence in epidemiology and other fields.^12^–^17^ In contrast, “hotspot” has long been used in genetics to refer to a highly polymorphic area in an organism's genome,^18^ where, for instance, cancer-causing mutations are likely to occur,^19^,^20^ or hypervariability allows a pathogen to evade the immune response.^21^ This usage is analogous to how “hotspot” is used when referring to areas of frequent emergence of novel pathogens.^22^ However, even within these broad categories, definitions can be unclear and subtle differences can have important implications.

## Hotspots as Areas of Elevated Disease Occurrence or Risk

In infectious disease epidemiology, “hotspot” is frequently used to refer to areas of elevated disease burden or high transmission efficiency. The term has gained traction in the study of malaria, where it has been used to describe groups of small areas (often less than ½ km^2^) with elevated incidence,^23^,^24^ similarly sized units of elevated transmission intensity,^2^,^25^ larger spatial units of elevated incidence (e.g., districts),^26^ and even countries with a high burden of disease.^1^ The term has been used for other diseases across spatial scales; including to designate neighborhoods with more efficient cholera transmission,^27^or countries where a high proportion of tuberculosis cases are multidrug resistant.^28^ Often the choice of spatial scale is driven by practical considerations. Data may be only available at one geographic scale,^26^–^28^ or there may be a specific scale at which relevant policies or interventions are implemented. There are other, more principled approaches. Algorithms such as SaTScan use statistical approaches to identify the spatial extent of hotspots.^29^ Bousema and others used disease biology to describe their geographic scale, defining malaria hotspots as areas smaller than the dispersal range of vector mosquitoes where the basic reproductive number (*R*_0_) is higher than surrounding areas.^2^

The difference between hotspots as areas of elevated incidence or prevalence versus elevated transmission efficiency is subtle but important. Whether the two measures lead us to identify the same areas as hotspots depends on the disease and how incidence and prevalence are measured. For instance, frequent malaria infection reduces the severity of infections^30^ so an area with intermittent transmission could have more symptomatic cases than one with frequent transmission.^24^ However, if incidence is measured based on the frequency of asymptomatic parasitaemia,^23^ or symptomatic infection in young (and more likely previously uninfected) children,^25^ then high incidence areas should also correspond to areas of elevated transmission. Depending on the timescale, similarly counterintuitive results may occur when comparing the distribution of disease in endemic and epidemic contexts. For example, cholera generally transmits with higher efficiency in Bangladesh than in Zimbabwe, which experiences epidemics every 3–5 years. However, if one were to look only at overall incidence of cholera between those two countries in 2008–2009, one might be led to erroneously conclude that Zimbabwe was more of a cholera hotspot due to the large epidemic that occurred that year.^31^ Hotspots as areas of elevated transmission intensity have received attention because more efficient control may be achieved by targeting these areas,^2^,^3^ especially if a single area appears to sustain transmission.^24^ For example, this has been promoted as one potential approach for the optimal employment of limited oral cholera vaccine supplies.^27^ Various factors have been shown to create transmission hotspots, including overcrowding,^32^ poverty,^33^ lack of adequate water, sanitation, and hygiene infrastructure,^34^ and geographic clusters of “core groups” that drive the transmission of STIs.^35^ Features that define the hotspot will dictate the development of appropriate control measures. The implications of such heterogeneous underpinnings of transmission provide a further argument for clarity, as the misspecification of hotspots based on differential causes may result in poorly designed, misguided, and potentially costly control efforts or missed opportunities for targeting limited resources.

Still, targeting hotspots is not a panacea for control. In outbreaks, generalized interventions or those targeted at “coldspots” may prove more effective, particularly if implemented late in response to epidemics, since the local epidemics in hotspots may have run their course by the time interventions can be put in place.^36^ Preventive vaccination strategies targeting hotspots may also be suboptimal if an intervention (e.g., vaccine) efficacy is incomplete and the transmission efficiency in the hotspot is sufficiently high so that even with high intervention coverage, close to 100% of the population will ultimately be infected in the hotstpot.^36^,^37^ Even with a proactive strategy and a nonleaky vaccine, the benefits could be larger in nonhotspot areas if transmission can be interrupted in these areas but not in hotspots (i.e., the reproductive number cannot be driven below one in the hotspot but can elsewhere).

## Areas Frequent for Disease Emergence or Reemergence

Hotspot can also refer to an area with a high risk for infectious disease emergence or reemergence. This common usage has captured the public's imagination in books such as *The Hot Zone*.^38^ Its origins are in ecology, where “hotspot” describes an area of high biodiversity.^39^ Hotspots of disease emergence are usually designated on broader spatial scales than those of elevated prevalence or transmission: southeast Asia is often referred to as an emerging infectious disease hotspot,^40^ as is West Africa,^41^ or even the entire African continent.^42^ While there have been attempts to map disease emergence at finer spatial scales,^43^ the goal has not necessarily been to identify highly localized disease emergence hotspots. Critical to defining emergence hotspots is the definition and identification of emergence events. Jones and others defined an emergence event as the first reported case of a new infectious disease in a human population.^43^ Others have considered each separate spillover event or zoonotic disease outbreak as an emergence,^42^ while others also include the foci of reemerging diseases such as cholera, yellow fever, and typhoid as hotspots.^44^

## Temporal and Spatial Characteristics of Hotspots

Hotspots of any type do not necessarily remain stable over time, but their temporal characteristics are rarely discussed. Exceptions include Bejon and others,^23^ who showed that some groups of homesteads in rural Kenya remain malaria transmission hotspots for years, whereas others, particularly those identified based on febrile malaria rather than parasitemia, lasted only months. Understanding the stability of hotspots over time is crucial for guiding disease-control strategies, otherwise we may end up chasing one former hotspot after the next, missing the true high risk areas.

## Conclusion

Hotspot is an evocative term that is often used in a casual and imprecise manner to spark interest in a paper or topic. Recently, the term has found increased use in policy and practice documents,^6^–^8^,^45^ guiding decisions on resource allocation and disease-control strategies. Although in some documents “hotspot” has a more specific definition (e.g., U.S. President's Malaria Initiative 2013^7^), others use the term with little clarification. Given the multiplicity of meanings, it may be best to forgo using this evocative term and use a more well-defined synonymous term instead. In many cases, alternative terms are more precisely defined in policy documents, such as malaria transmission foci^46^ or HIV-hyperendemic countries.^47^ However, when hotspot is used, we suggest that such uses require a more descriptive explanation of the criteria by which that hotspot is defined to prevent misinterpretation and confusion.

We recommend that the meaning of a “hotspot” be made explicit by use of an appropriate modifier such as: “burden hotspot,” to denote areas of elevated disease prevalence or incidence; “transmission” or “risk hotspot,” to denote areas of elevated transmission efficiency or a higher risk of disease acquisition; and “emergence hotspot,” to denote areas with an increased probability of disease emergence or reemergence. Furthermore, when geographic scale is important, a precise, data-based definition of spatial extent and characteristics should be provided. Table 1 provides a summary of the various definitions of hotspot common in the literature, key citations, and suggested alternative or clarifying terms to reduce the risk of confusion and simplify policy recommendations.

Spatial heterogeneity in disease processes is a growing field of study. The identification of hotspots of all types can play an important role in research, policy, and practice, particularly in resource planning, allocation, and implementation in response to infectious diseases. However, public health, like all fields, is subject to fads in concepts and terminology. Hotspot is an evocative term for important concepts, but a useful term should not be rendered useless by imprecision or overuse. Policy makers and public health researchers should be sensitive to its multitude of uses within the health sciences, and take care in their own use of the term.

## Figures and Tables

**Table 1:** Overview of definitions for different types of “hotspots”

Definition	References	Suggested term and alternatives
An area of elevated transmission efficiency (i.e., elevated reproductive number, *R*)	2,3,27,36	Transmission hotspot, transmission foci
An area with a high frequency of emergence or reemergence of diseases or drug-resistant strains.	40–44	Emergence hotspot
An area of elevated disease incidence or prevalence or a geographic cluster of cases	23,24,26,28	Burden hotspot, high burden country/province/and so on, hyperendemic region, case cluster
